# Hospital Reform Association

**Published:** 1898-01-22

**Authors:** 


### HOSPITAL REFORM ASSOCIATION.

The first annual meeting of the Hospital Reform Association was held on Wednesday afternoon at 20, King William Street, Strand. Dr. Ward Cousins presided, and at the commencement of the proceedings there were four members present, including the chairman and the hon. secretary (Mr. Garrett Horder). At a later stage the total increased to nine, among whom were Colonel Montefiore, Dr. Snape, Dr. Sibley, and Mr. T. R. Atkinson.

The report, which was read by the Hon. Secretary, stated that the Council, in presenting their first annual report, had good reason for congratulating the members on the progress made in the promotion of hospital reform. Though the Association was only instituted on October 1st, 1896, it had organised and taken part in a considerable number of meetings in London and the provinces. "Your Council," the report proceeded, "would like to point out that at no previous time has the question been so much discussed by the medical as well as by the lay Press, and they are of opinion that the public are beginning to realise the necessity of bringing about some tangible reforms. Your Council feel that the real work of the Association is only now commencing, and they would earnestly beg the members to influence their professional and non-professional friends to attach themselves to it. In conclusion, your Council would like to point out that it was entirely owing to the efforts and the representations of the Association that an Investigation Committee was appointed to report on the Manchester medical charities. An effort is being made at Brighton to appoint a committee, which, your Council regret to add, has not, so far, met with much encouragement from the managers of the hospitals in that town. Your Council would also like to mention that the managers of the large general hospitals in the metropolis have appointed a committee to draw up a scheme for the better administration of medical relief in the out-patient and casualty departments."

According to the financial report the Association had received £67 and spent the same amount. The liabilities amounted to £50, and there was a balance of £5 in hand. The reading of this statement brought Mr. T. R. Atkinson on his feet, who strongly protested against the Association being in debt. "I have," he said, "been a member of several medical associations which have failed through want of attention to the financial side. I think we should not have launched into these expensive meetings if we had not the means to pay our way."

Colonel Montefiore also expressed his regret that the Association was in debt, but he had no doubt the hon. secretary would be able to say he had some plan to meet the deficiency.

The Hon. Secretary stated that so far only 25 out of 140 members had paid their subscriptions for the current year, so there was a good deal to come from this source. Mr. Atkinson, however, still protested, and pointed to the handful of persons who were present at the meeting, whereupon Dr. Snape explained that London was suffering from influenza, and that the general practitioners were very busy grappling with the scourge. Personally he thought the money had been very well spent, and he did not think they need worry themselves about the debt. Still Mr. Atkinson was not satisfied, for upon the re-election of the Council and officers he again expressed the hope that debt would in future be avoided. This elicited from the Chairman an announcement to the effect that the British Medical Association had that day discussed the question of giving a donation to the Hospital Reform Association, but before taking definite action they desired to obtain a statement respecting the finances and the work of the Association.

The ordinary business having been disposed of, the Hon. Secretary proceeded to read a paper on "Hospital Reform," which he summed up under three heads as follows: 1. That in the casualty department of our large general hospitals only cases of urgent importance should be attended to. 2. That in the out-patient department patients bringing notes from medical men should have a prior claim to treatment. That a resident physician should be appointed, whose duty it shall be to see all out-patients in the first instance and select those that require immediate treatment and decide which do not require hospital treatment. That after patients have received "first aid" their circumstances shall be inquired into by a competent officer. That the honorary medical officer shall not be required to treat more than 20 new cases at one sitting. 3. That all in-patients, with the exception of cases of accident or of great urgency, should be recommended for treatment by medical men, and that before being admitted their circumstances should be inquired into by an officer specially retained for that purpose.

A committee consisting of Dr. Sibley, Dr. Snape, Dr. Isambard Owen, and Dr. J. A. Dauber was appointed to consider the paper in detail and to bring up a report at a subsequent meeting.

The proceedings then terminated.

